# Intrahepatic Glissonean Approach for Robotic Anatomical Liver Resection of Segment 7 Using the Saline-Linked Monopolar Cautery Scissors (SLiC-Scissors) Method: A Technical Case Report With Videos

**DOI:** 10.7759/cureus.38470

**Published:** 2023-05-03

**Authors:** Takahisa Fujikawa, Yusuke Uemoto, Taisuke Matsuoka

**Affiliations:** 1 Surgery, Kokura Memorial Hospital, Kitakyushu, JPN

**Keywords:** saline-linked cautery scissors, hepatocellular carcinoma, liver parenchymal transection, anatomical s7 subsectionectomy, robotic liver resection

## Abstract

Anatomical hepatectomy of segment 7 (S7) is technically difficult due to its difficult accessibility. Here, we present our experience of robotic anatomical S7 subsectionectomy of the liver employing the saline-linked cautery scissors (SLiC-Scissors) technique. After the right lobe was fully mobilized, dissection of the Glissonean pedicle and hepatic venous branch of S7, as well as the liver parenchymal transection, were safely performed using the SLiC-Scissors method. Despite its technological complexity, the intrahepatic Glissonean approach for robotic anatomical S7 subsectionectomy of the liver employing the SLiC scissors method is safe and efficient.

## Introduction

Due to its limited accessibility and anatomical position close to the right hepatic vein (RHV), anatomical liver resection of segment 7 (S7) is technically difficult [1,2]. In both open and laparoscopic surgery, some methods of anatomical S7 hepatectomy with an intrahepatic Glissonean approach have been documented [1,3,4]. However, due to the inherent limits of laparoscopic procedures, such as difficulty in properly exposing the operative view and the limited degrees of devices [5], laparoscopic anatomical S7 hepatectomy appears to be complicated.

Robotic liver resections (RLRs) have gained widespread acceptance and a wider range of applications in recent years. Compared to traditional laparoscopy, RLRs have a number of advantages such as tools with seven degrees of freedom, steady three-dimensional vision, and tremor filtration [6-8]. Nonetheless, liver parenchymal transection is one of the most difficult steps of RLR due to a lack of equipment. A recent article described the unique technique of the saline-linked cautery scissors (SLiC-Scissors) method in RLR [9]. This method allows for simultaneous hemostasis and quick liver parenchymal dissection during RLR.

The current paper describes a case of laparoscopic anatomic liver resection of S7 via the intrahepatic Glissonean approach using the SLiC-Scissors method.

## Technical report

**Figure 1:**
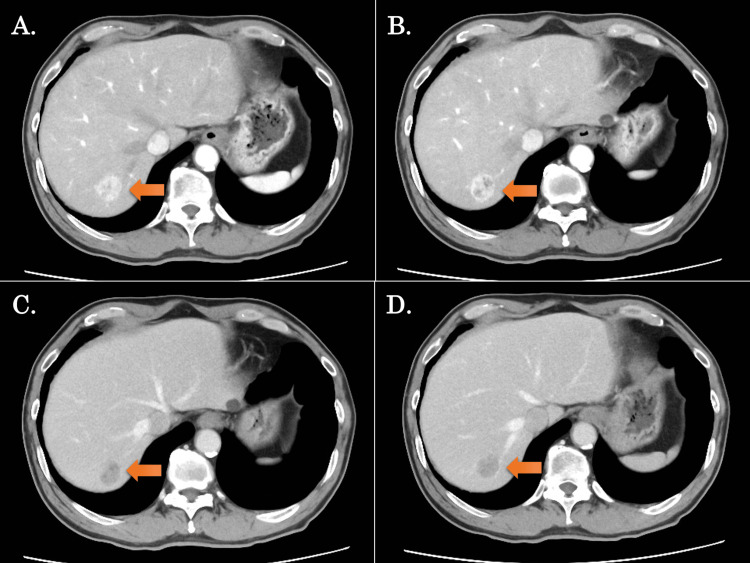
Preoperative CT images in the present case A-D show contrast-enhanced CT findings (arrows indicate the tumor); (A, B) The images of the early phase of contrast enhancement, (C, D) Images of the late phase of contrast enhancement. The tumor was located in segment 7, with a maximum diameter of 25 mm.

A 67-year-old male patient was diagnosed with hepatocellular carcinoma and was referred to our department for further examination and treatment. Preoperative imaging confirmed the presence of a tumor with a maximum diameter of 25 mm in S7 (Figure 1, Video 1). Liver functional reserve revealed normal function with Child-Pugh grade 1 (score 5) and an indocyanine green (ICG) retention rate at 15 min of 7.1%. For surgical intervention, a robotic anatomical S7 subsectionectomy of the liver via an intrahepatic Glissonean approach was scheduled.

**Video 1:** Preoperative images of the 3D construction from the contrast-enhanced CT The tumor was located in segment 7, which was adjacent to the major venous branch in S7 (V7).

Patient position and port placement

**Figure 2:**
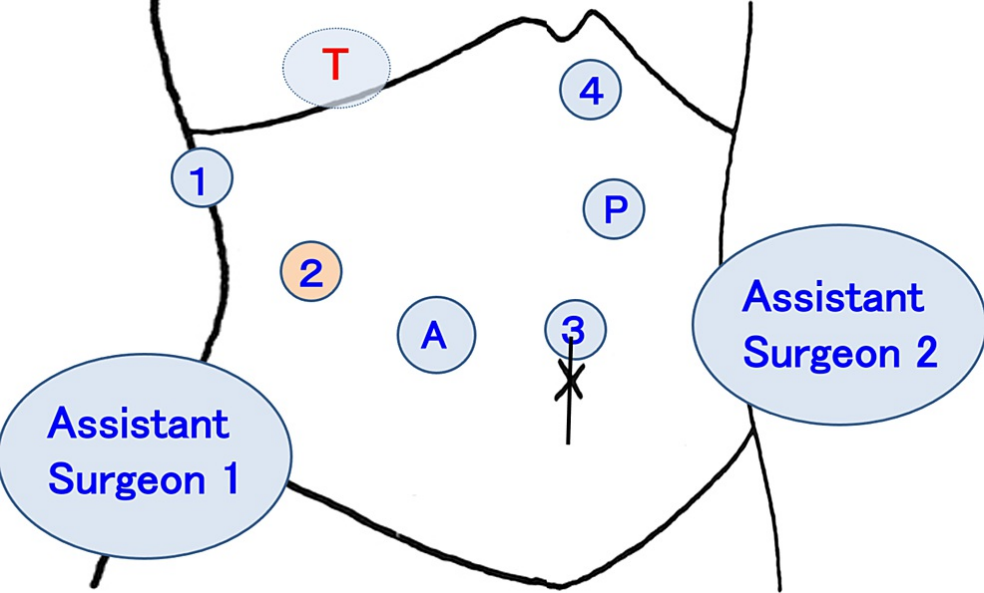
Trocar placement for robotic anatomical S7 subsectionectomy Five trocars were typically used for the procedure; robotic #1 port was placed at the right upper lateral side of the abdomen, #3 was placed above the umbilicus, #2 and a 12-mm assistant port were placed between #1 and #3, and #4 was placed at the left-sided epigastrium. The patient cart was rolled in and placed on the right cranial side of the patient. The first assistant surgeon stood on the patient's right side while the second assistant surgeon stood on the left side. T: the targeted lobe of the liver; A: a 12-mm trocar for the assistant surgeon; 1,2,3,4: the four robotic ports; P: Pringle maneuver site

The patient was placed in a lateral decubitus position with a slight left posterior tilt, in which the head side was raised 8-10 degrees. Using a flexed operating table, the right side of the abdomen was extended and fixed. The first trocar was inserted through an incision made on the top of the umbilicus, and intraabdominal pressure was maintained at 8-10 mmHg. The da Vinci® Xi Surgical System (Intuitive Surgical, Inc., Sunnyvale, CA) was used for the procedure. As for the robot ports, #1 was placed at the right upper lateral side of the abdomen, #3 was placed cranial to the umbilicus, #2 and a 12-mm assistant port were placed between #1 and #3, and #4 was placed at the left-sided epigastrium (Figure 2). On the patient's right cranial side, the patient cart was rolled in and set down.

Full mobilization of the right liver in RLR

**Figure 3:**
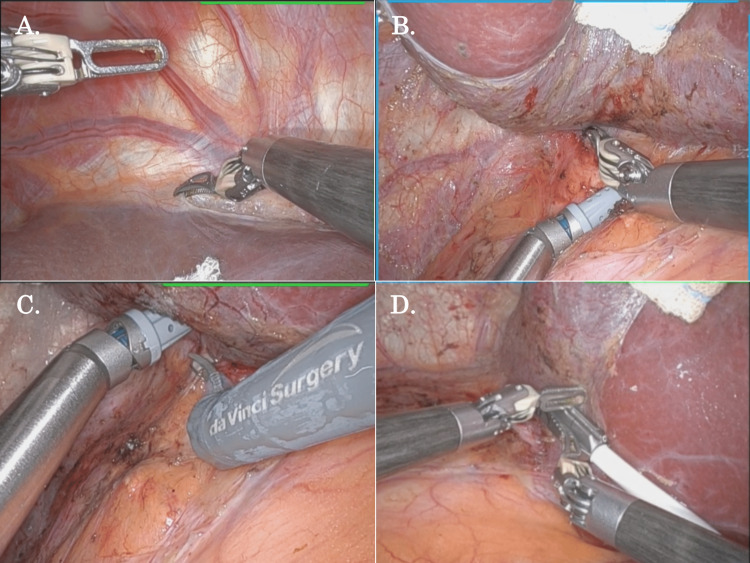
The process of full mobilization of the right liver in robotic anatomical S7 hepatectomy (A) After the round ligament was severed by laparoscopic coagulating shears, the falciform and right coronary ligaments were dissected using the double bipolar method. (B) The liver was mobilized from the retroperitoneum. (C) A dissection was made between the right adrenal gland and the liver, and the right adrenal vein was clipped and severed. (D) After the right liver was fully mobilized, the Glissonean pedicle in S7, the venous branch in S7, and the hepatic tumor were identified by intraoperative ultrasound and marked.

When the liver was mobilized, the double bipolar method was used [10], and the surgical field was secured by port-hopping of the camera (Figure 3, Video 2). The #1, #2, #3, and #4 robotic ports were primarily assigned to liver retraction (the third arm), the surgeon’s left hand, the endoscope, and the right hand, respectively. The #1, #2, and #4 arms were operated using Tip-up Forceps®, Fenestrated Bipolar Forceps®, and Maryland Bipolar Forceps®, respectively. After the extraperitoneal tourniquet system for the Pringle maneuver was placed and the round ligament was sealed and severed by laparoscopic coagulating shears (LCS; Ethicon, Cincinnati, Ohio), the falciform and right coronary ligaments were dissected. Dissection was continued until the right triangular ligament was severed (Figure 3A).

**Video 2:** The process of full mobilization of the right liver in robotic anatomical S7 hepatectomy

Subsequently, the liver was mobilized from the retroperitoneum to expose the inferior vena cava. In this step, the camera was hopping from port #3 to port #2 to directly visualize the surgical field, with Fenestrated Bipolar Forceps®, Maryland Bipolar Forceps®, and Tip-up Forceps® being placed in ports #1, #3, and #4, respectively. Tip-up Forceps® was used for liver retraction to stabilize the surgical field, and a dissection between the liver and retroperitoneum was made (Figure 3B). During the dissection, the right adrenal vein was clipped and severed (Figure 3C). After the right liver was fully mobilized, the Glissonean pedicle in S7 (G7), the venous branch in S7 (V7), and the hepatic tumor were identified by intraoperative ultrasound and marked (Figure 3D).

Liver parenchymal transection in RLR using the SLiC-Scissors method

**Figure 4:**
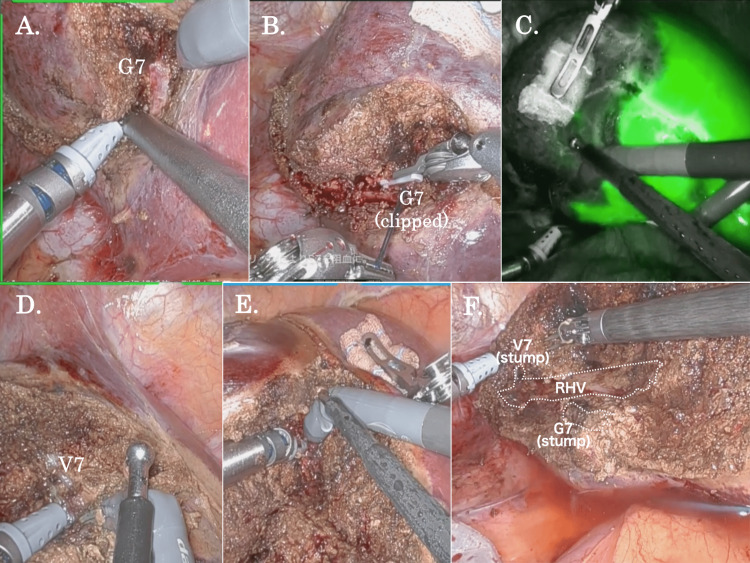
The process of liver parenchymal transection in robotic anatomical S7 hepatectomy using the SLiC-Scissors method (A) The root of the G7 was dissected by the water-jet scalpel. (B) G7 was ligated and clipped. (C) The clear demarcation line for S7 was confirmed by intraoperative ICG negative staining. (D) V7 was exposed, clipped, and severed. (E) ICG imaging with the robotic Firefly system was used to correct the displacement of the cut plane as appropriate. (F) After the RHV was exposed from cranially to caudally, liver parenchymal transection was completed. SLiC-Scissors: saline-linked electrocautery scissors, S7: segment 7 of the liver, G7: Glissonean pedicle in S7, ICG: indocyanine green, V7: the venous branch in S7, RHV: right hepatic vein

During liver parenchymal transection in RLR, the SLiC-Scissors method was used (Figure 4, Video 3) [9]. Briefly, the camera was placed in the #2 port, and the EndoWrist Suction Irrigator® (Intuitive Surgical, Inc.) and monopolar curved scissors were placed in the #1 and #3 ports, respectively. Using monopolar scissors, the parenchymal transection was advanced while hemostasis with low-temperature heat coagulation of the superficial layer of the dissection surface was simultaneously conducted by dripping saline drops from the assistant side. Vascular structures with a diameter greater than 2 mm were clipped, ligated, and divided robotically or by the assistant surgeon utilizing LCS.

**Video 3:** Liver parenchymal transection in robotic anatomical S7 hepatectomy using the SLiC-Scissors method SLiC-Scissors: saline-linked electrocautery scissors

Using the SLiC-Scissors method, hepatic parenchymal transection adjacent to the G7 was carried out, and the root of the G7 was dissected using a water-jet scalpel (Erbejet® 2, Erbe Elektromedizin GmbH, Tuebingen, Germany), ligated, and clipped (Figures 4A, 4B). Subsequently, the area of S7 became ischemic, and the clear demarcation line was observed, which was confirmed by intraoperative ICG negative staining (Figure 4C). The tumor staining by preoperative ICG administration was also detected.

Following that, parenchymal transection was performed to expose V7 and RHV, and V7 was encircled, clipped, and severed (Figure 4D). After sufficient parenchymal transection around the G7, the G7 was severed by LCS. ICG imaging with the robotic Firefly system was used to appropriately correct the cut plane's displacement (Figure 4E). After the RHV was exposed from cranially to caudally to control direct bleeding from the RHV, liver parenchymal transection was completed (Figure 4F).

The Pringle maneuver was performed as necessary, and the blood flow was interrupted twice (30 min) before the liver transection was completed. The total operative time and docking time were 367 min and 267 min, respectively, and the total blood loss was minimal with no intraoperative transfusion required. The postoperative course was uneventful, and the patient was discharged on postoperative day 7.

## Discussion

The intrahepatic Glissonean approach using the SLiC-Scissors method for robotic anatomical S7 subsectionectomy of the liver is described in the current article. Using this strategy, rapid and secured dissection around G7 and RHV can be achieved during RLR. Additionally, it might be done without increasing the risk of bile leakage or bile duct stenosis in the right posterior section due to heat damage. Consequently, the current approach can deliver a safe and time-efficient approach for RLR.

According to the available data, the main limitation of RLR is the transection of the liver parenchyma. Recently, a novel approach for robotic liver parenchymal transection utilizing the SLiC-Scissors method has been described [9], which combines the benefits of the existing instruments for laparoscopic and robotic surgery. The current method is based on the Kyoto University-style liver parenchymal transection procedure during open liver resection [11], in which low-temperature heat coagulation (up to 100 degrees Celsius) is applied to the superficial layer by saline-linked bipolar electrocautery. The SLiC-Scissors method in RLR also utilizes superficial low-temperature heat coagulation via monopolar cautery scissors with droplets of saline from the assistant side [9]. As this method allows the tip of the scissors to remain clean using saline, fast, thin-layered dissection and hemostasis are simultaneously achieved.

Concerning the technique for laparoscopic anatomical S7 hepatectomy, various approaches have been reported such as the Glissonean approach to the G7 from the hepatic hilum [12,13], the intrahepatic Glissonean approach [1,3], or the caudate lobe first approach [14,15]. Except for the intrahepatic Glissonean approach, however, these approaches require additional and unnecessary dissection around the liver hilum and/or caudate lobe and might cause incidental biliary complications and bleeding.

In the case of a robotic approach for anatomical S7 hepatectomy, the disadvantages of RLR include loss of tactile sensation and the risk of damage to important vessels and parenchyma from inadvertent movement [8]. For this reason, we introduced the intrahepatic Glissonean approach to avoid additional dissection around the liver hilum and/or caudate lobe. Moreover, when the SLiC-Scissors method combined with a water-jet scalpel is utilized during RLR, the liver parenchymal transection around the major Glissonean pedicles and major hepatic veins can be approached more quickly and comfortably [9]. Thus, we think the intrahepatic Glissonean approach using the SLiC-Scissors method is one of the preferred techniques for robotic anatomical S7 subsectionectomy of the liver.

Some papers suggest that intercostal ports are useful for laparoscopic approaches to the posterosuperior part of the liver [2]. However, the so-called EndoWrist® function, which enables the handling of the instrument tips in seven degrees of freedom, can be utilized in RLR. Combined with stable three-dimensional visualization and tremor filtration with motion scaling capacity, the robotic procedures allow for a comfortable and accurate dissection even for anatomical S7 hepatectomy without using intercostal ports.

## Conclusions

We report our experience with robotic anatomical S7 subsectionectomy of the liver. Although robotic anatomical S7 subsectionectomy is a technically demanding procedure, the intrahepatic Glissonean approach using the SLiC-Scissors method is safe and practical. This approach allows for the meticulous dissection and management of the intrahepatic Glissonean pedicle and major hepatic vein in S7.

## References

[1] Okuda Y, Honda G, Kobayashi S, Sakamoto K, Homma Y, Honjo M, Doi M (2018). Intrahepatic Glissonean pedicle approach to segment 7 from the dorsal side during laparoscopic anatomic hepatectomy of the cranial part of the right liver. J Am Coll Surg.

[2] Inoue Y, Suzuki Y, Fujii K (2017). Laparoscopic liver resection using the lateral approach from intercostal ports in segments VI, VII, and VIII. J Gastrointest Surg.

[3] Takagi K, Kuise T, Umeda Y, Yoshida R, Teraishi F, Yagi T, Fujiwara T (2020). Laparoscopic liver resection of segment seven: a case report and review of surgical techniques. Int J Surg Case Rep.

[4] Machado MA, Herman P, Machado MC (2003). A standardized technique for right segmental liver resections. Arch Surg.

[5] Lee JW, Choi SH, Kim S, Kwon SW (2020). Laparoscopic liver resection for segment VII lesion using a combination of rubber band retraction method and flexible laparoscope. Surg Endosc.

[6] Croner RS, Perrakis A, Hohenberger W, Brunner M (2016). Robotic liver surgery for minor hepatic resections: a comparison with laparoscopic and open standard procedures. Langenbecks Arch Surg.

[7] Chen PD, Wu CY, Hu RH (2017). Robotic major hepatectomy: is there a learning curve?. Surgery.

[8] Liu R, Wakabayashi G, Kim HJ (2019). International consensus statement on robotic hepatectomy surgery in 2018. World J Gastroenterol.

[9] Fujikawa T, Uemoto Y, Matsuoka T, Kajiwara M (2022). Novel liver parenchymal transection technique using saline-linked monopolar cautery scissors (SLIC-Scissors) in robotic liver resection. Cureus.

[10] Kikuchi K, Suda K, Shibasaki S, Tanaka T, Uyama I (2021). Challenges in improving the minimal invasiveness of the surgical treatment for gastric cancer using robotic technology. Ann Gastroenterol Surg.

[11] Yamamoto Y, Ikai I, Kume M (1999). New simple technique for hepatic parenchymal resection using a Cavitron Ultrasonic Surgical Aspirator and bipolar cautery equipped with a channel for water dripping. World J Surg.

[12] Cheng KC, Yeung YP, Hui J, Ho KM, Yip AW (2011). Multimedia manuscript: laparoscopic resection of hepatocellular carcinoma at segment 7: the posterior approach to anatomic resection. Surg Endosc.

[13] He JM, Zhen ZP, Ye Q, Mo JQ, Chen GH, Peng JX (2020). Laparoscopic anatomical segment VII resection for hepatocellular carcinoma using the Glissonian approach with indocyanine green dye fluorescence. J Gastrointest Surg.

[14] Morise Z (2016). Laparoscopic liver resection for posterosuperior tumors using caudal approach and postural changes: a new technical approach. World J Gastroenterol.

[15] Li H, Honda G, Ome Y, Doi M, Yamamoto J, Muto J (2019). Laparoscopic extended anatomical resection of segment 7 by the caudate lobe first approach: a video case report. J Gastrointest Surg.

